# Synergetic enhancement in the reactivity and stability of surface-oxide-free fine Al particles covered with a polytetrafluoroethylene nanolayer

**DOI:** 10.1038/s41598-020-71162-z

**Published:** 2020-09-03

**Authors:** Dong Won Kim, Kyung Tae Kim, Dong Uk Lee, Soo-Ho Jung, Jihun Yu

**Affiliations:** 1 3D Printing Materials Research Center, Korea Institute of Materials Science, 797 Changwondaero, Seongsan-gu, Changwon, Gyeongnam 51508 Republic of Korea; 2 Department of Industrial Chemistry, Pukyong National University, 45 Yongsoro, Nam-gu, Busan 48513 Republic of Korea

**Keywords:** Energy science and technology, Materials science

## Abstract

Surface oxide (Al_2_O_3_) of reactive fine aluminum (Al) particles for solid fuels, propellants, and brazing materials often restricted oxidative performance, though the passivation film acts to protect Al particles from exploding. Here, we report fine Al particles fully covered with a polytetrafluoroethylene (PTFE) layer instead of an Al_2_O_3_ film on the surface. This advance is based on the introduction of strong Al–F bonds, known to be an alternative to the Al–O bonds of surface oxides. The DSC results on the PTFE-coated Al particles exhibit higher reactive-exothermic enthalpy energy (12.26 kJ g^−1^) than 4.85 kJ g^−1^ by uncoated Al particles. The artificial aging test of the PTFE layer on the Al particles show long-time stability to the external circumstance compared to those by Al_2_O_3_. The activation energy for oxidation was investigated from cyclic voltammetry assessment and the measured peak potentials of the anode curve for PTFE/Al (− 0.45 V) and uncoated Al (− 0.39 V) are achieved, respectively. This means that the PTFE layer is more stable against a sudden explosion of Al particles compared to Al_2_O_3_. These results are very useful given its capability to control both the reactivity and stability levels during the oxidation of Al particles for practical applications.

## Introduction

Compared to other metals, aluminum (Al) releases a higher amount of energy (31.05 kJ g^−1^) when oxidized^1–3^. This is especially true when the Al is in powder form due to its maximized surface area in this form. As such, it can be used as a source of energy in solid fuels, propellants, and brazing materials^2–7^. However, as Al spontaneously undergoes surface passivation in the atmosphere, a 2–10-nm-thick oxide layer generally forms^8,9^. Such an oxide layer is known to be thermodynamically stable such that the oxidative efficiency decreases at certain temperatures or under certain conditions, as it retards the reaction between internal Al and the surrounding oxygen molecules^6,10–14^. However, when the surface oxide layer is removed, there is a risk of sudden explosion due to rapid oxidation. To control the threshold of reactivity, many researchers have developed processes that increase the reactivity of Al particles using organic/inorganic compounds^14–17^. For example, Guo et al.^15^ improved the thermal properties that the exhibit a lower oxidation onset and a higher enthalpy by coating the Al nanopowder with carbon. Bunker et al.^16^ showed that oleic acid-capped Al nanoparticles with oxide film have stability in air and easy reactivity in water.

Materials such as fluoropolymers^10,18–22^, epoxides^23^, iron^24^, palladium^9^, and nickel^25–27^ have shown advantageous properties for ignition and combustion when they are mixed with Al powders. In particular, it is well known that fluoropolymers are chemically and thermally more stable than other organic compounds, but they have also attracted substantial attention as an ideal material for passivation^28–30^. Considering that a large amount of energy (664 kJ mol^−1^) is released when F atoms react with Al to form AlF_3_ as compared to Al_2_O_3_, fluoropolymers are highly promising as oxidative additives with which to realize high reactivity of Al particles^31^. It is expected that macromolecules such as polytetrafluoroethylene (PTFE, –(CF_2_–CF_2_)_n_–), which are composed exclusively of fluorine functional groups (with the exception of the backbone), will offer many benefits, including high oxidative reactivity.

However, because most previous studies use a simple mixing process rather than a powder coating method to add fluoropolymers, including PTFE, enhancing the efficiency of the energy release caused by a surface oxide has remained limited^10,19–21,32^. Although some studies report coating methods^18^, there are no results about surface treatments that can safely remove oxide materials on fine Al particles. That is, technology that offers the direct coating of fine Al particles has yet to be developed due to experimental difficulties with both the coating of a full-fluorine-based polymer and removing the surface oxide to reveal the pure Al. Thus, the development of highly stable and highly reactive Al particles at the same time is a key issue related to the practical use of energetic powders.

Here, we report surface-oxide-free Al particles covered with PTFE materials obtained from a spontaneous coating process of PTFE nanoparticles just after the removal of the oxide layer from the Al surface. The fine Al particles used in this study have diameters of 5 µm on average, as this size is appropriate considering the reactivity and stability of this material compared to conventional Al particles of a few tens of μm and a few hundreds of nm in size. Direct contact at the PTFE/Al interface is confirmed through cross-sectional transmission electron microscope (TEM) images, and the thermodynamic behavior of the coated Al particles compared to uncoated particles is clearly revealed through a thermogravimetric analysis. Furthermore, the resistance to oxidation of the Al powder due to the PTFE coating was analyzed electrochemically, with the stability of the particles verified through comparisons with a passivation layer of Al_2_O_3_.

## Results and discussion

### Preparation and microstructure of PTFE coated fine Al particles

**Figure 1:**
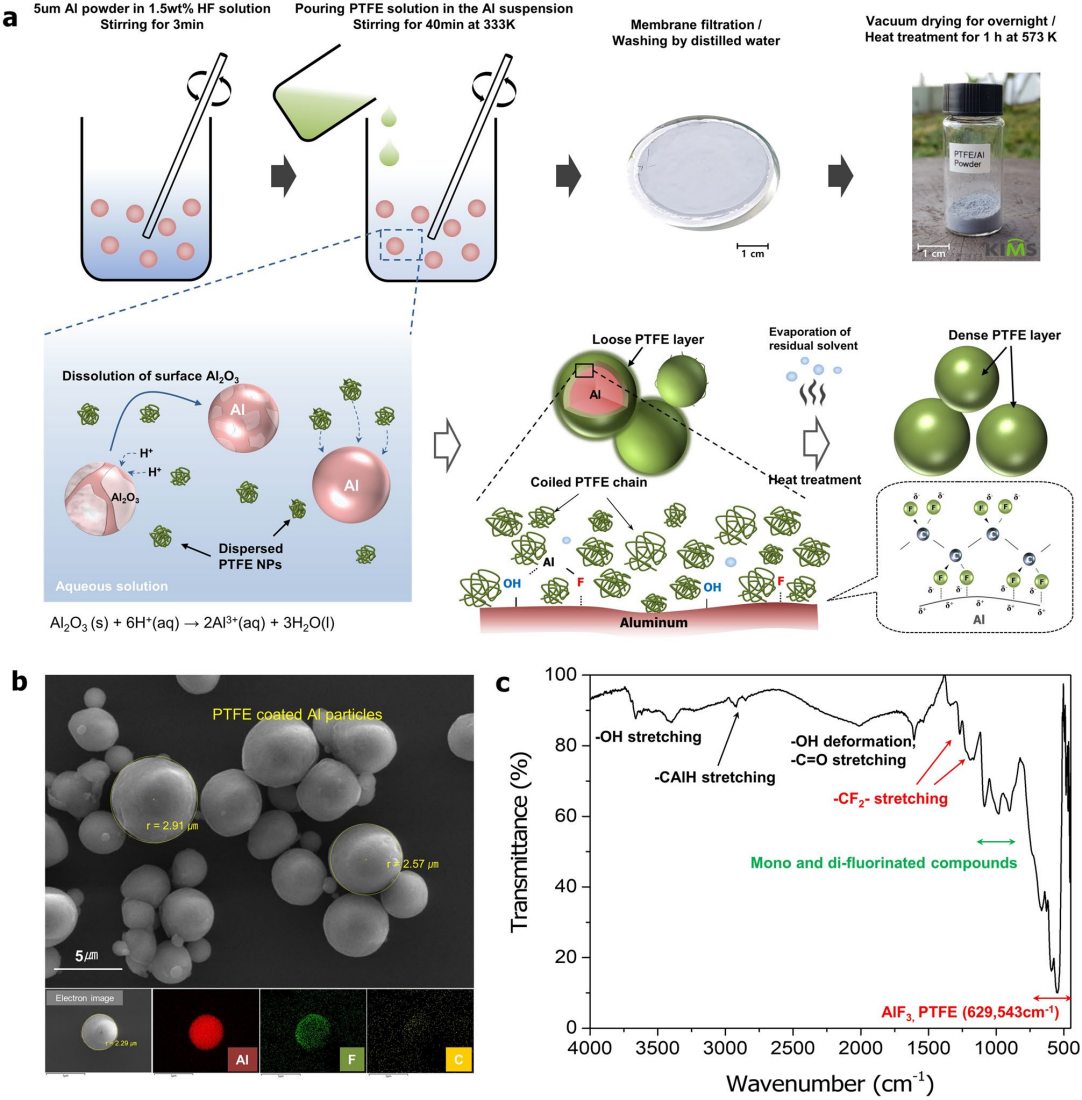
(**a**) Conceptual illustration showing the synthesis of the PTFE-coated Al particles via fluoride adsorption on the surface in the experimental process, (**b**) SEM and EDS mapping images of the synthesized PTFE/Al particles, and (**c**) transmission FT-IR spectrum of the synthesized PTFE/Al powder.

Figure 1a shows the synthetic process of Al particles densely covered with PTFE materials via chemical routes using suspended PTFE nanoparticles. First, the surface oxide layer on the Al particles is decomposed into AlF_3_ and H_2_O by the addition of a hydrofluoric acid (HF) solution, expressed as the following reaction (1).

1 $$ {\text{Al}}_{{2}} {\text{O}}_{{3}} {\text{(s)}} + {\text{6HF(aq)}} \to {\text{2AlF}}_{{3}} {\text{(aq)}} + {\text{3H}}_{{2}} {\text{O(l)}} $$

Once the oxide layer was removed, the fluorine attached to PTFE and isolated from HF initially adsorbed onto the exposed pure Al surface. Because F atoms have the highest electronegativity for Al compared to other elements such as oxygen, the surface must be preferentially covered by fluorine-based materials. Some AlF_3_ produced during this process was simultaneously dissolved and hydrated due to its high solubility in an aqueous solution. The as-formed PTFE layer may not be fully densified due to the free volume caused by the remaining solvent among the nanoparticles. Therefore, the prepared PTFE/Al powder was heated to produce a densely packed coating layer. Figure 1b shows surface SEM images and EDS results of the synthesized PTFE/Al particles. In spite of the etching process, the size of the Al particles of approximately 5 µm is mostly unchanged. The distributions of the F and C atoms shown in the EDS results of the coated Al particles indicate that the PTFE materials are clearly coated, covering the surfaces of the particles. Figure 1c, which compares the FT-IR spectra of the PTFE/Al particles and PTFE molecules, clearly indicates that their peaks in the 800–1,300 cm^−1^ region are in good agreement with those of aliphatic fluorinated compounds^33,34^. The peaks at 1,273 cm^−1^ and 1,194 cm^−1^ indicate typical –CF_2_– functional group stretching of the PTFE. Other FT-IR peaks of the PTFE/Al particles were found at 3,675, 3,415 and 1604 cm^−1^; these can be attributed to the formation of Al–OH stretching, hydrogen-bonded OH stretching by intramolecular motion and OH deformation vibration, respectively. These results show that the PTFE materials were spontaneously adsorbed onto the surface of the Al by surface fluoridation.

**Figure 2:**
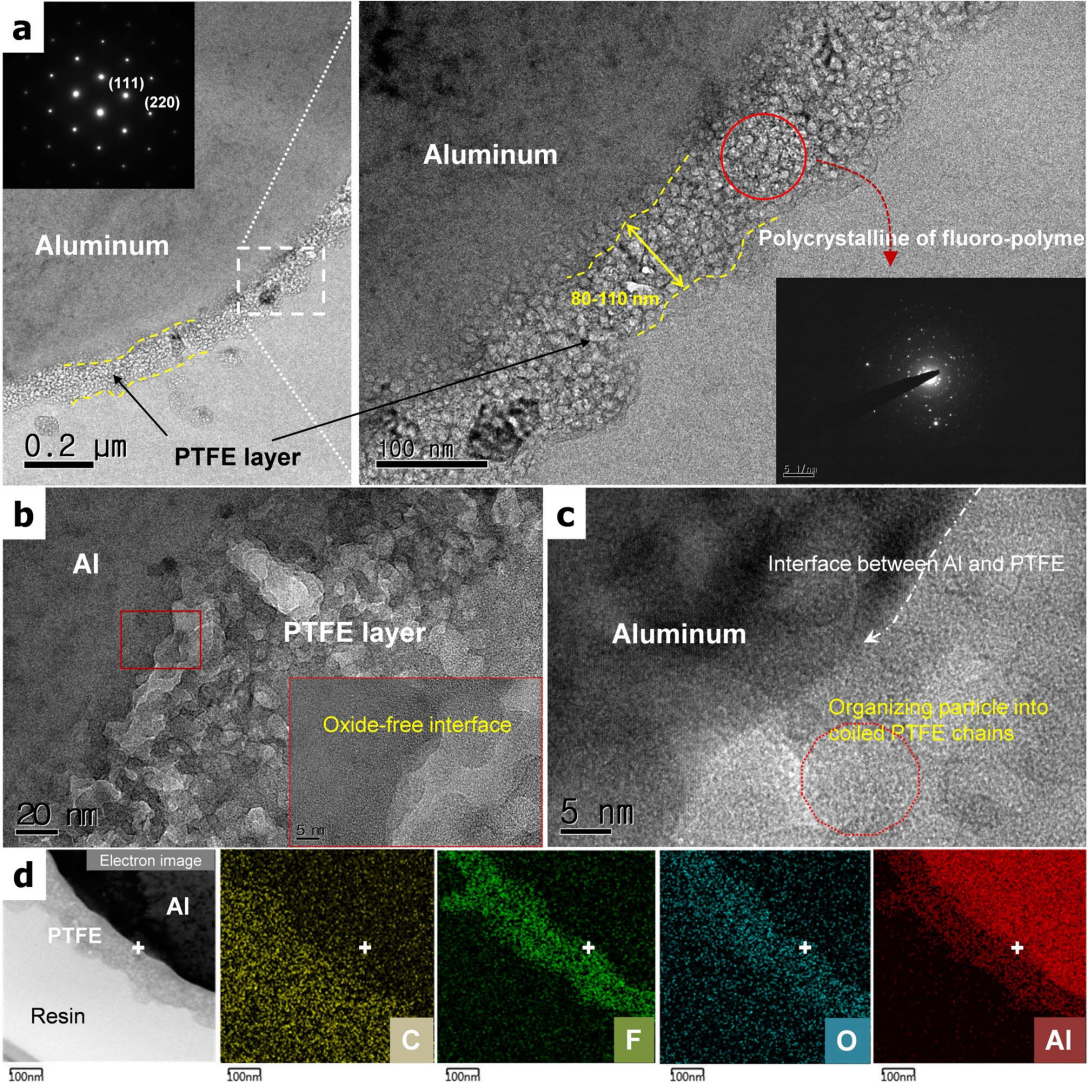
(**a**) Cross-sectional TEM images of a PTFE/Al particle, (**b**,**c**) enlarged TEM image of the PTFE/Al interface region, and (**d**) EDS results showing the distributions of carbon, fluorine, oxygen, and aluminum atoms.

Figure 2 shows a cross-sectional FE-TEM image of a PTFE/Al particle produced by a focused ion beam (FIB). The measured thickness of the PTFE layer is approximately 100 nm. The selected-area electric diffraction (SAED) pattern of the PTFE layer indicates a polycrystalline structure composed of many nano-sized PTFE chains. In general, organic polymers show high permeability to oxygen and water molecules due to their chain structure compared to an inorganic Al_2_O_3_ layer^35^. An increase in the thickness of the coating layer considering the permeability of moisture or oxygen is considered to be an effective means of achieving stability matching that of passivated Al_2_O_3_ film thinner than 5 nm. Therefore, it has been suggested that the thickness of PTFE as a coating layer should be at least 100 nm to maintain the surface of the pure Al consistently. This value is based on our previous results with polyvinylidene fluoride (PVDF)/Al particles^22^. Figure 2b shows a TEM image of the PTFE/Al interface, showing an absence of oxide materials. As shown in the enlarged TEM image in Fig. 2c, the coating layer near the interface consists of coiled PTFE chains 10 nm in size, also without an oxide layer between the Al and the PTFE. The EDS results in Fig. 2d present the distributions of carbon, fluorine, oxygen, and Al atoms in the particles. The PTFE coating is clearly confirmed by the dense C and F atomic layers on the Al particles. Some oxygen atoms present within the PTFE layer are from the Al–OH formation step during the etching process.

### Exothermic reactivity of uncoated Al and PTFE/Al powders

**Figure 3:**
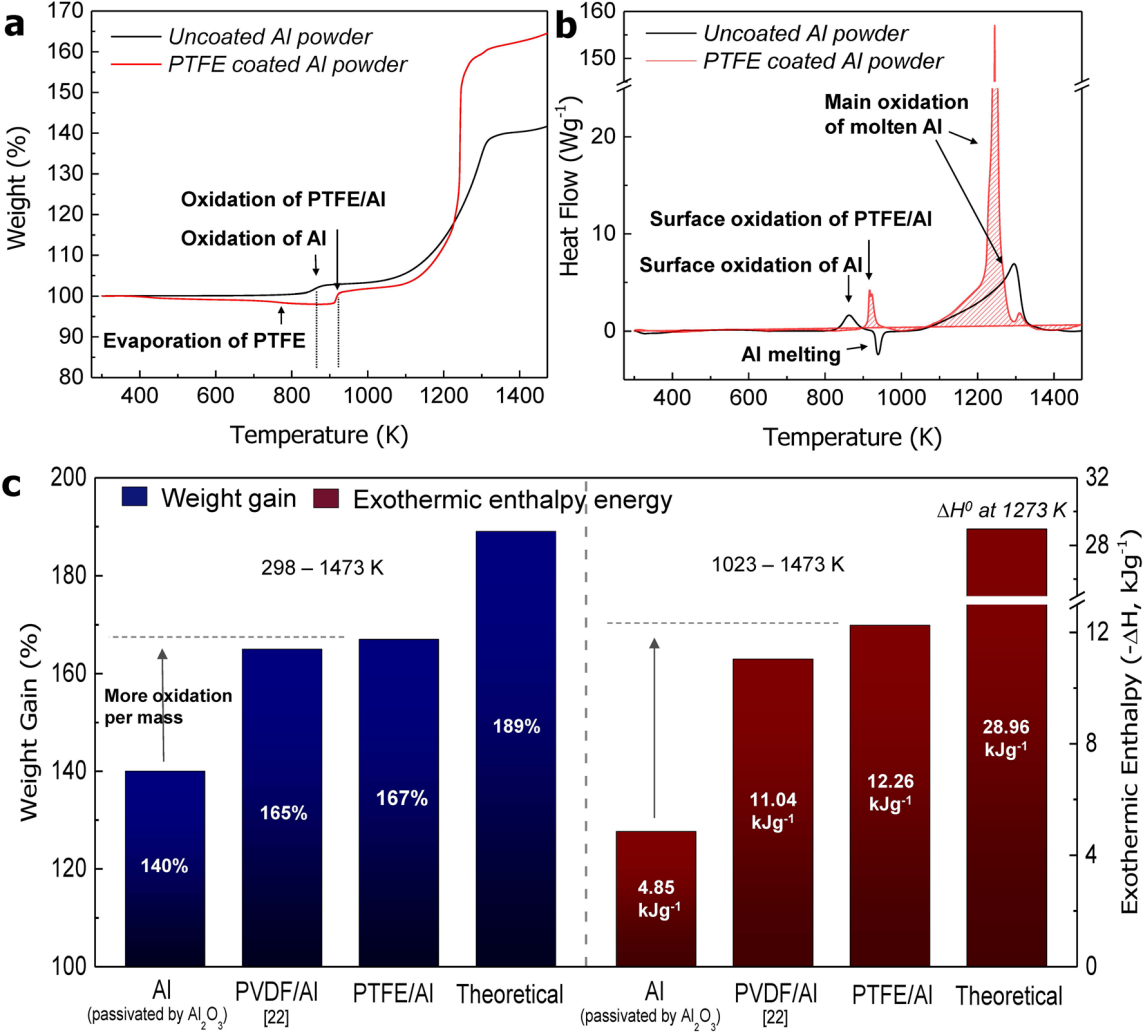
(**a**,**b**) Comparison of the TGA/DSC curves between the PTFE/Al and the uncoated Al powders in an air atmosphere, and (**c**) comparison of weight gains and exothermic enthalpy energy levels.

Figure 3a,b show the thermal analysis results of the uncoated Al powder and the PTFE/Al powder between 298 and 1,473 K. The PTFE/Al powder was shown to be stable at room temperature, but there was a weight reduction of approximately 3% from the temperature of approximately 620 K due to thermal decomposition. Surface oxidation of the uncoated Al and PTFE/Al powders occurred at 873 K and 923 K, respectively. The main oxidation step, which occurs at temperatures exceeding 1,100 K, proceeded more rapidly for the PTFE/Al powder than for the Al powder. In the differential scanning calorimetry (DSC) curve shown in Fig. 3b, the uncoated Al powder clearly show an exothermic peak due to surface oxidation at 860 K and an endothermic peak at 933 K due to melting. Conversely, the PTFE/Al powder was found to undergo somewhat delayed surface oxidation owing to the relatively thick PTFE layer. The wide peaks of both powders observed in the range of 1,050–1,350 K were caused by the main oxidation of the internal Al, which is in a molten state in this range.

In Fig. 3c, the quantitative weight and exothermic enthalpy of Al and PVDF/Al from the TGA/DSC profiles are compared. The PVDF/Al data from our previous research is included for a comparison with the PTFE/Al powder under identical conditions. The weight of Al increased by 140%, and the exothermic enthalpy in the temperature range of 1,023 K to 1,473 K was found to be 4.85 kJ g ^−1^. In addition, the PTFE/Al powder shows an improved weight gain of 167%, and the enthalpy was determined to be 12.26 kJ g^−1^. These values are somewhat higher than those of a previously synthesized PVDF/Al powder^22^ (see Fig. S1 in the Supplementary Information), as the number of F atoms in PTFE present during the pyrolysis process exceeds that associated with PVDF (–(CH_2_–CF_2_)_n_–). Specifically, the formation of many fluorine compounds in PTFE is beneficial as it synergistically provides enhanced reactivity to the oxidation processes occurring on the Al surface because complex surface reactions such as the formation of the AlF_3_ phase will inevitably occur at the PTFE/Al interface compared to those with the Al_2_O_3_–passivated Al powder. The theoretical enthalpy is 28.96 kJ g^−1^ at 1,273 K (details of Equation S1 in the Supplementary Information), and this value was utilized in the comparison of the results of the uncoated Al and PTFE/Al powders.

**Figure 4:**
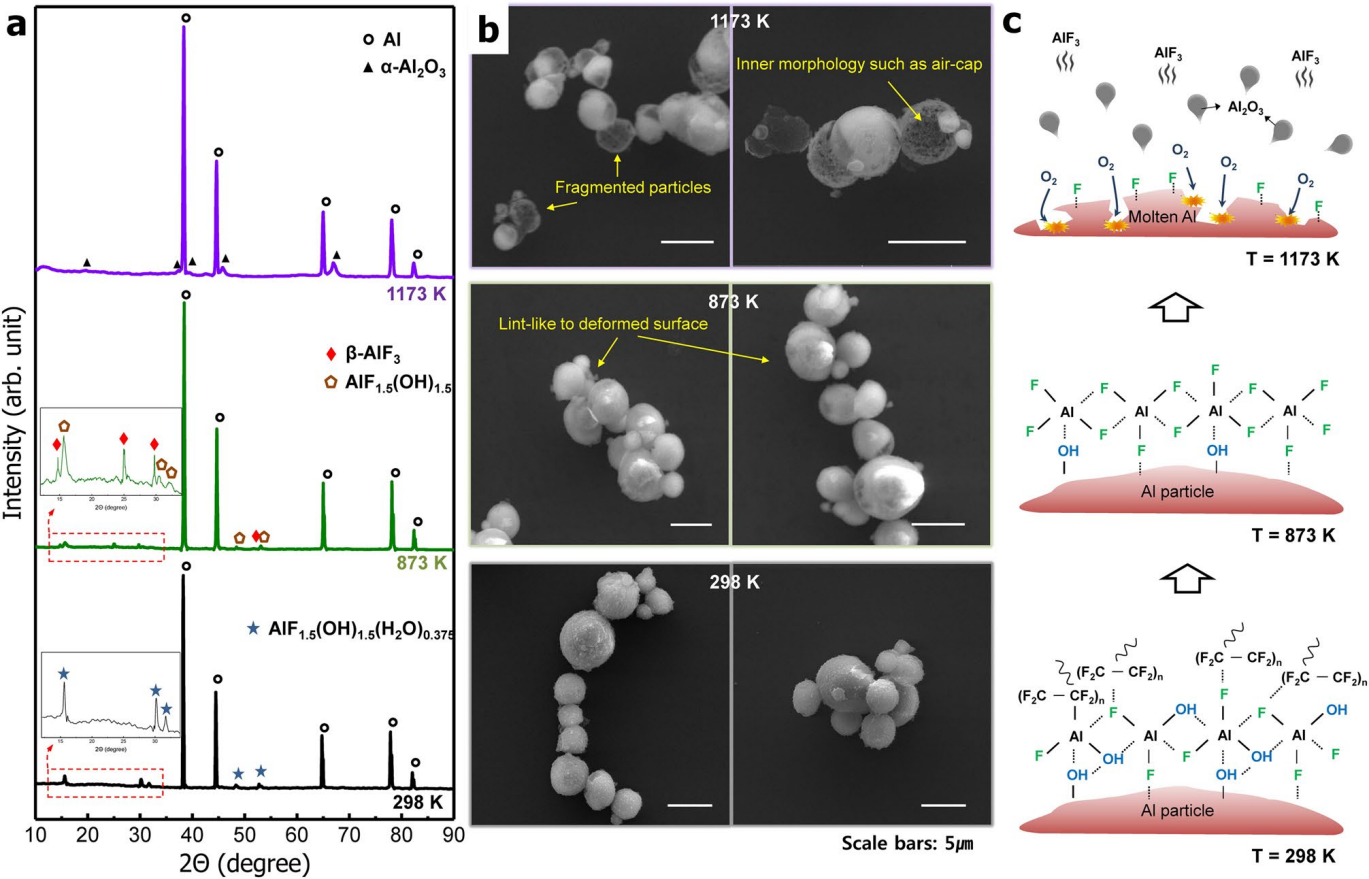
(**a**) X-ray diffraction patterns, (**b**) SEM images and (**c**) schematic of the fluorine compounds formed on the surfaces of the PTFE/Al powder after heat treatments at 298 K, 873 K and 1,173 K.

The PTFE/Al powder was heat-treated at temperatures of 873 K and 1,173 K in an air atmosphere in order to confirm the complex reactions caused by the fluorine atoms. Figure 4a,b compare the XRD patterns and SEM images of the PTFE/Al powder with three different heat treatment temperatures of 298 K, 873 K and 1,173 K. Figure 4c is a schematic of the fluorine compounds and the reaction occurring on the surfaces of the Al particle in each temperature section. The XRD pattern at 298 K indicates that the main peaks of the PTFE/Al powder, except for aluminum, correspond to AlF_1.5_(OH)_1.5_(H_2_O)_0.375_. The heat-treated PTFE/Al powder at 873 K show peaks at 2θ = 15.8°, 30.4°, and 31.8°, indicating the formation of AlF_1.5_(OH)_1.5_, and the additional peaks observed at 2θ = 14.8°, 25.0° and 29.7° represent the formation of β-AlF_3_^36,37^. This outcomes demonstrate that aluminum fluoride initially forms before the aluminum oxide does according to the stronger bonding capabilities of Al-F (664 kJ mol^−1^) compared to those of Al–O (512 kJ mol^−1^)^31^. A lint-like deformed surface of the PTFE/Al particles was observed in the SEM images at 873 K. At 1,173 K, where the bulk of oxidation takes place, only patterns of aluminum and Al_2_O_3_ phases were identified, with broken Al particle fragments also observed. These results show that the particle shell can easily be broken by the thermal expansion of molten aluminum due to fluorine compounds which form on the surface, as we reported earlier paper. Thus, the reactivity of fine Al particles can be enhanced via the direct coating with PTFE materials.

### Stability and reactivity of PTFE/Al powder

**Figure 5:**
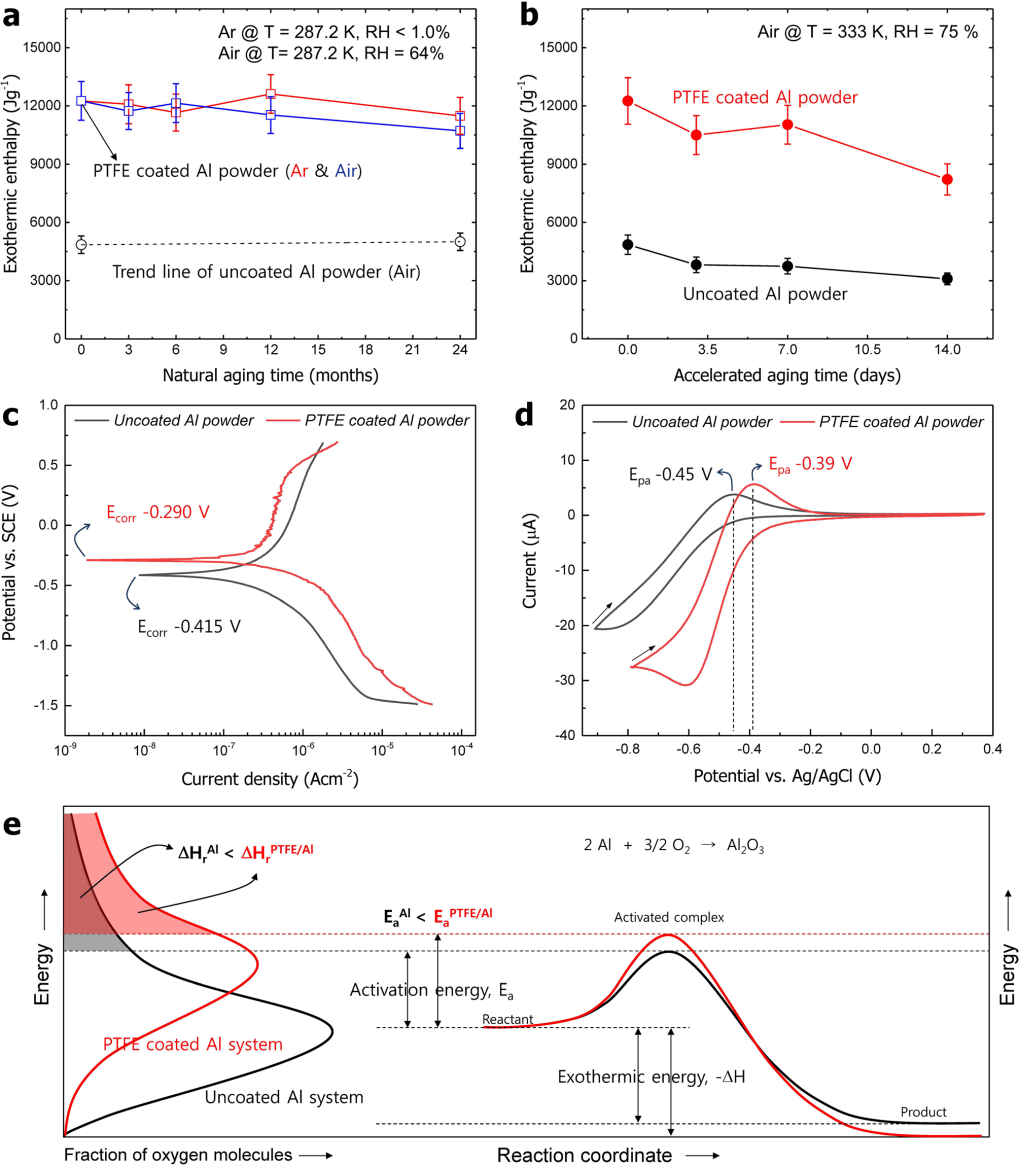
Comparison of the exothermic enthalpy change as a function of time from (**a**) natural aging and (**b**) accelerated aging tests, (**c**) potentiodynamic polarization and (**d**) cyclic voltammetry curves of PTFE/Al and uncoated Al powders, and (**e**) summarized conceptual illustration of the reactivity and stability for oxidation in both systems.

Furthermore, the stability of the synthesized PTFE/Al powder was characterized by aging tests and an electrochemical analysis. As shown in Fig. 5a, the PTFE/Al powder was classified into two experimental groups, one stored under air and the other in an argon atmosphere. The uncoated Al powder with a dense oxide film underwent no change in the enthalpy value for more than 24 months (see Fig. S2 for details). Similarly, the PTFE/Al powder stored in an argon atmosphere also exhibited a very stable enthalpy value during the test period. Regarding the PTFE/Al powder stored in air, although the enthalpy value decreased slightly to 10.71 kJ g^−1^ after 24 months, it is difficult to postulate that the performance clearly deteriorated because this sample did not show a great difference from the experimental tolerance range (12.26 ± 0.98 kJ g^−1^). Therefore, we conducted an artificial accelerated aging test under extreme conditions. The accelerated aging test was performed for two weeks at 333 K under a relative humidity of 75%, as shown in Fig. 5b. For both the uncoated Al and the PTFE/Al powders, the enthalpy value tended to decrease after three days, with the corresponding values reduced to 3.09 kJ g^−1^ and 8.21 kJ g^−1^, after 14 days (see Fig. S3). When both conditions of natural aging and accelerated aging are converted to the mole fraction of water by a psychrometric calculator, the outcomes are approximately 0.01 and 0.15, respectively. The respective absolute humidity levels are 10,000 ppm and 150,000 ppm, indicating that the accelerated aging test was conducted in a very humid atmosphere of approximately 15 times higher compared to that of the natural aging test. In other words, re-passivation can proceed at a certain threshold of moisture even if PTFE is densely coated onto the Al particles. Nevertheless, the fact that the PTFE/Al powder has an exothermic enthalpy value more than twice that of the uncoated Al powder indicates that the PTFE/Al interface and the coating layer are stably maintained without any severe passivation by oxygen.

As shown in Fig. 5c, the measured corrosion potentials (E_corr_) of the uncoated Al and the PTFE/Al powders are − 0.415 V and − 0.290 V, respectively. Because this is a potential at which electrons are emitted from the surface of the material, it is interpreted that more activation energy is required to oxidize the PTFE/Al powder compared to the uncoated Al powder. The corrosion current (I_corr_) induced by the Tafel extrapolation method showed that the PTFE/Al powder has a value of 0.079 µAcm^−2^, higher than that of the uncoated Al powder of 0.074 µA cm^−2^ (see Fig. S4). In the cyclic voltammetry assessment, the inflection point of the anode curve peak represents the potential at which oxidation occurs at the working electrode. As shown in Fig. 5d, the measured peak potentials of the anode curve (E_pa_) are − 0.45 V for uncoated Al and − 0.39 V for the PTFE/Al powders. These results clarify that the activation energy for the oxidation of the PTFE/Al powder is higher than of the uncoated Al powder.

The reactivity and stability for the oxidation of both PTFE/Al and uncoated Al particles can be summarized by a conceptual illustration using the activation energy and Boltzmann distribution curves, as shown in Fig. 5e. The higher activation energy peak means that the PTFE layer introduced in this study provides superior stability against a sudden explosion of Al particles compared to the surface oxide layer of Al_2_O_3_. As shown in the comparison of the Boltzmann curves, the large surface area of pure Al under the PTFE layer provides numerous reaction sites for oxygen molecules, resulting in a high exothermic enthalpy energy release at an elevated temperature beyond the activation energy. Additionally, it should be noted that the activation energy of both powders can change after surface oxidation. Such a prediction of the change in the activation energy is plotted in Supplementary Figure S5 using the Arrhenius equation.

## Conclusion

The PTFE-coated Al powder with an average particle size of 5 µm is synthesized via an in-situ process with an acidic aqueous solution. The PTFE coating layer is chemically bound to and is directly in contact with the Al surface. The amount of exothermic enthalpy energy of the Al powder is significantly enhanced by the introduction of a PTFE coating instead of Al_2_O_3_. The improved reactivity is analyzed due to complex reactions induced by fluorine atoms on the surface of the pure Al core. Al fluoride compounds prevent the Al surface from readily undergoing oxygen-passivation according to the principle of strong Al-F bonds. Aging test results here confirmed that the stability of the PTFE layer remains constant until the particles undergo severe oxidation due to an ultra-high-humidity condition. Therefore, the stability of the PTFE/Al powder is greater than that of the uncoated Al powder at room temperature, and the former releases more energy during oxidation. The stability and reactivity, which exist in a trade-off relationship, are synergistically controlled by the PTFE coating to replace the oxide film of Al particles. These fine Al particles covered with the PTFE nanolayer developed in this study provide many opportunities for the realization of high-performance energetic materials.

## Methods

### Chemicals and materials

Al powder having spherical particles with an average size of 5 µm was used. The surface oxide was approximately 6.6 nm thick (see Fig. S6). A commercial PTFE dispersion (TF 5035Z) with an average size of around 200 nm purchased from 3 M Dyneon. Hydrofluoric acid (HF, J. T. Baker, 48.0–51.0 wt%) was used to remove the alumina layer which surrounded the Al particle.

### Preparation of PTFE-coated Al powder

A diluted PTFE solution was prepared by adding 2.0 g of the commercial PTFE dispersion to 38.0 g of the deionized water. The diluted solution was sonicated to be finely dispersed into nano-sized particles at 323 K for 30 min.

An acidic etching solution (40.0 g) was prepared using 38.8 g of distilled water with 1.2 g of 48.0–51.0 wt% hydrofluoric acid, after which 1.0 g of Al powder was slowly pouring dispersed in the this solution. Approximately 3 min after bubbling was observed, 20 mL of the diluted PTFE solution was poured into the etched Al suspension, followed by mechanical stirring of about 400 rpm at 333 K for 40 min. The synthesized PTFE/Al powder was membrane filtered and washed with least five times with distilled water and EtOH, and then vacuum-dried at 333 K for 24 h. Finally, heat treatment of PTFE/Al powder was performed in the electric furnace at 573 K for 1 h under argon atmosphere (see at the FT-IR spectra in Fig. S7).

### Accelerated aging test

About 1.0 g of the PTFE/Al powder prepared in the same batch process is divided into three samples and spread widely on a dish with a sieve. Each sample of experimental group was stored for 3 days, 7 days and 14 days in a thermo-hygrostat chamber maintained at 75% relative humidity and 333 K. The powders recovered in the chamber were dried in a vacuum oven for 24 h, and then analyzed thermal properties.

### Characterization

Microstructural characterization and thermal analysis on the coated powders were conducted on the basis of our previous studies^22,25^. The surface morphology of the powders was characterized by field-emission scanning electron microscopy (FE-SEM, MIRA II LMH, Tescan). The local composition and elemental distribution on the particle surface were analyzed by FE-SEM with EDS^22,25^. And the chemical bonding of the PTFE coated on the Al particle was analyzed by Fourier transform infrared spectroscopy-attenuated total reflectance (FTIR-ATR, Nicolet iS5, Thermo Scientific)^22^. The phases of the powders were analyzed by XRD (D/Max-2500VL, Rigaku International Co.). TGA and DSC (Q600, TA Instruments) were conducted at a heating rate of 10 K min^−1^ from 298 to 1,473 K in the atmosphere^22,25^. The enthalpies of the powders subjected to thermal analysis were calculated from the peak area under the DSC curves. The DSC equipment was calibrated for temperature and enthalpy using indium as a standard material according to the ASTM E967 and E968 guidelines^25^.

A cross-sectional specimen of PTFE/Al powder for TEM observation was prepared using a FIB (NOVA200, FEI Inc.) lift-off technique (see Fig. S8). A field-emission transmission electron microscope operated at 200 kV (JEM-2100F, JEOL) was used for the microstructural analysis of the PTFE/Al interface^25^.

In order to check the stability to external circumstance, the potentiodynamic polarization tests were performed on an electrochemical workstation (PARSTAT 2,273, Princeton Applied Research) with the three-electrode cell system. Carbon rod and saturated calomel electrode (SCE) were used as counter electrode and reference electrode, respectively. Both Al and PTFE/Al powders dispersed in acetonitrile (Sigma-Aldrich, 98%) were deposited on glassy carbon electrode with 3 mm diameter and used as a working electrode. The polarization curves of working electrodes were tested at a scanning rate of 5 mV s^−1^ in 3.5 wt% NaCl solutions. In addition, cyclic voltammetry measurements were carried out on the electrochemical workstation (VersaSTAT3, Princeton Applied Research) with a conventional three-electrode system using an Ag/AgCl as reference electrode, Pt coil as counter electrode, and the glassy carbon as the working electrode. The cyclic voltammetry was measured at a scan rate of 10 mV s^−1^ in 0.1 M tetrabutylammonium tetrafluoroborate (TBABF, Aldrich, 99%) solutions. And 0.001 M of ferrocene was used as a calibration material for the reference electrode.

## Supplementary information

Supplementary file1.

## Data Availability

The data that support the findings of this study are available from the corresponding author upon a reasonable request.
