# Supplementary material for: Synergetic enhancement in the reactivity and stability of surface-oxide-free fine Al particles covered with a polytetrafluoroethylene nanolayer
Source: Sci Rep. 2020 Sep 3;10:14560. doi: 10.1038/s41598-020-71162-z (PMC7471686; doi:10.1038/s41598-020-71162-z)
Supplement: Supplementary file 1 — Supplementary file1. [file 41598_2020_71162_MOESM1_ESM.doc]

Supplementary Information

Synergetic Enhancement in the Reactivity and Stability of Surface-Oxide-Free Fine Al particles Covered with a Polytetrafluoroethylene Nanolayer

Dong Won Kim,^†^ Kyung Tae Kim,^*,†^ Dong Uk Lee,^‡^ Soo-Ho Jung^†^, Jihun Yu^†^

^†^ 3D Printing Materials Research Center, Korea Institute of Materials Science, 797 Changwondaero, Seongsan-gu, Changwon, Gyeongnam 51508, Republic of Korea

^‡^ Department of Industrial Chemistry, Pukyong National University, 45 Yongsoro, Nam-gu, Busan, 48513, Republic of Korea

^*^E-mail: ktkim@kims.re.kr


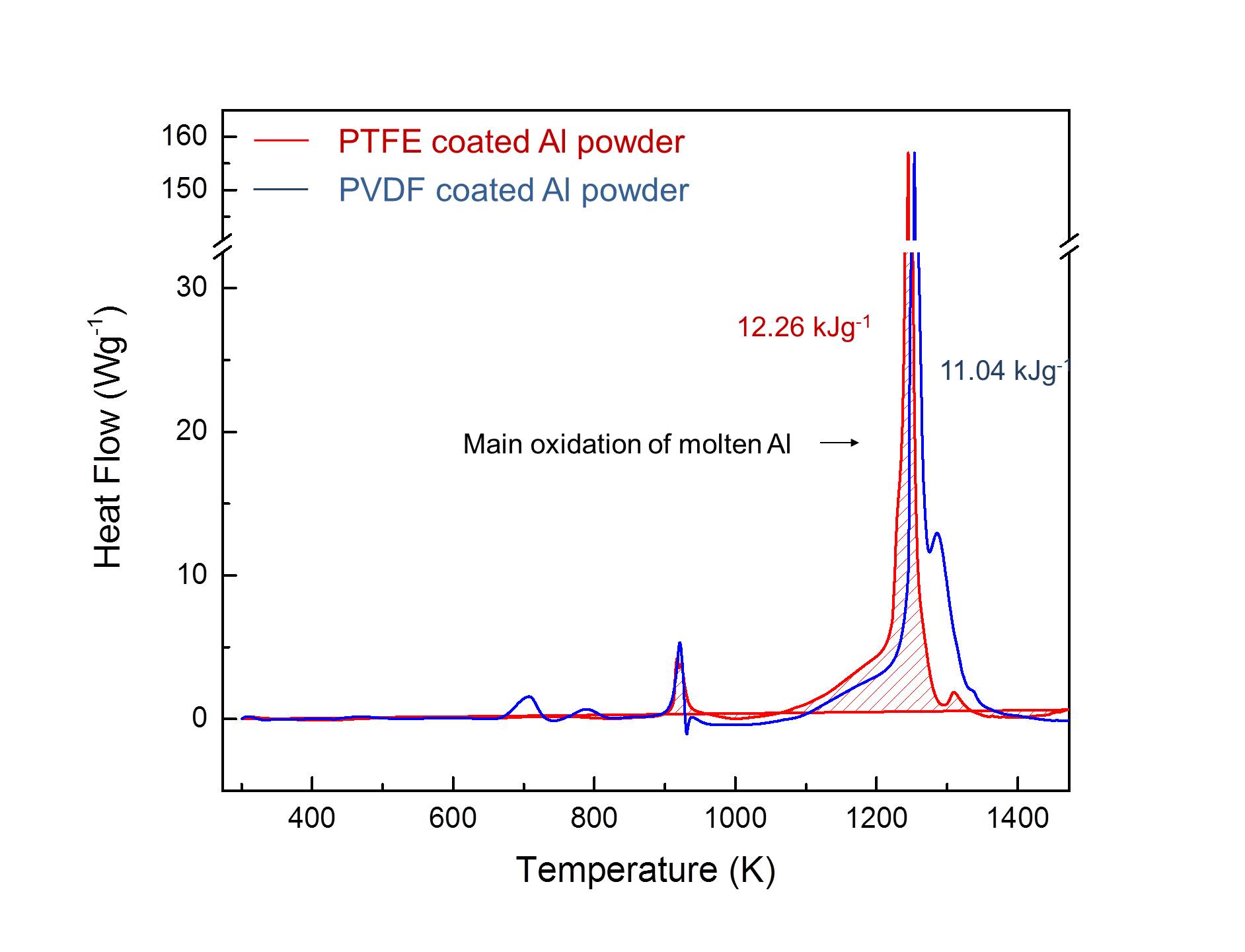


Figure S1. DSC curves of the PTFE/Al and PVDF/Al powders under air atmosphere.

Theoretical weight gain is calculated by 189 % when active Al except surface oxide is fully transformed into Al_2_O_3_. Our PTFE/Al and uncoated Al particles show meaningful change in heat flow in the temperature range of 1023-1473 K. Thus, following equation (S1) are used for formation enthalpy value of Al_2_O_3_ at 1273 K.


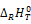
 =
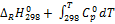
 (S1)


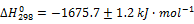
,
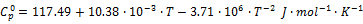


The calculated theoretical reaction enthalpy of Al was 28.96 kJg^-1^ at 1273 K, which was compare with experimental results in PTFE/Al, PVDF/Al and uncoated Al powders in the manuscript.


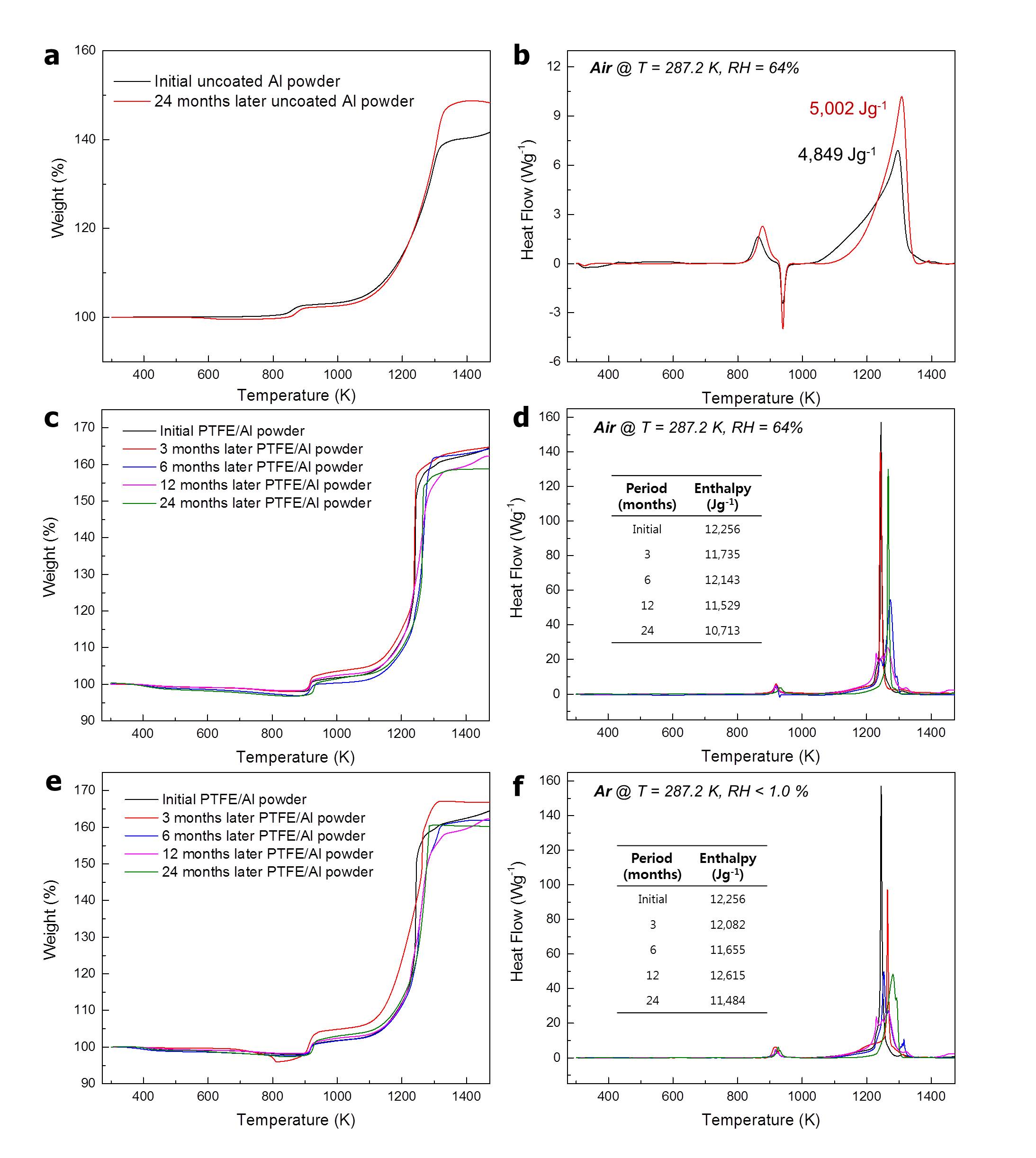


Figure S2. Comparison of the TGA/DSC curves of (a-b) uncoated Al powder under air atmosphere and PTFE/Al powder under (c-d) air and (e-f) argon atmosphere over natural aging time.


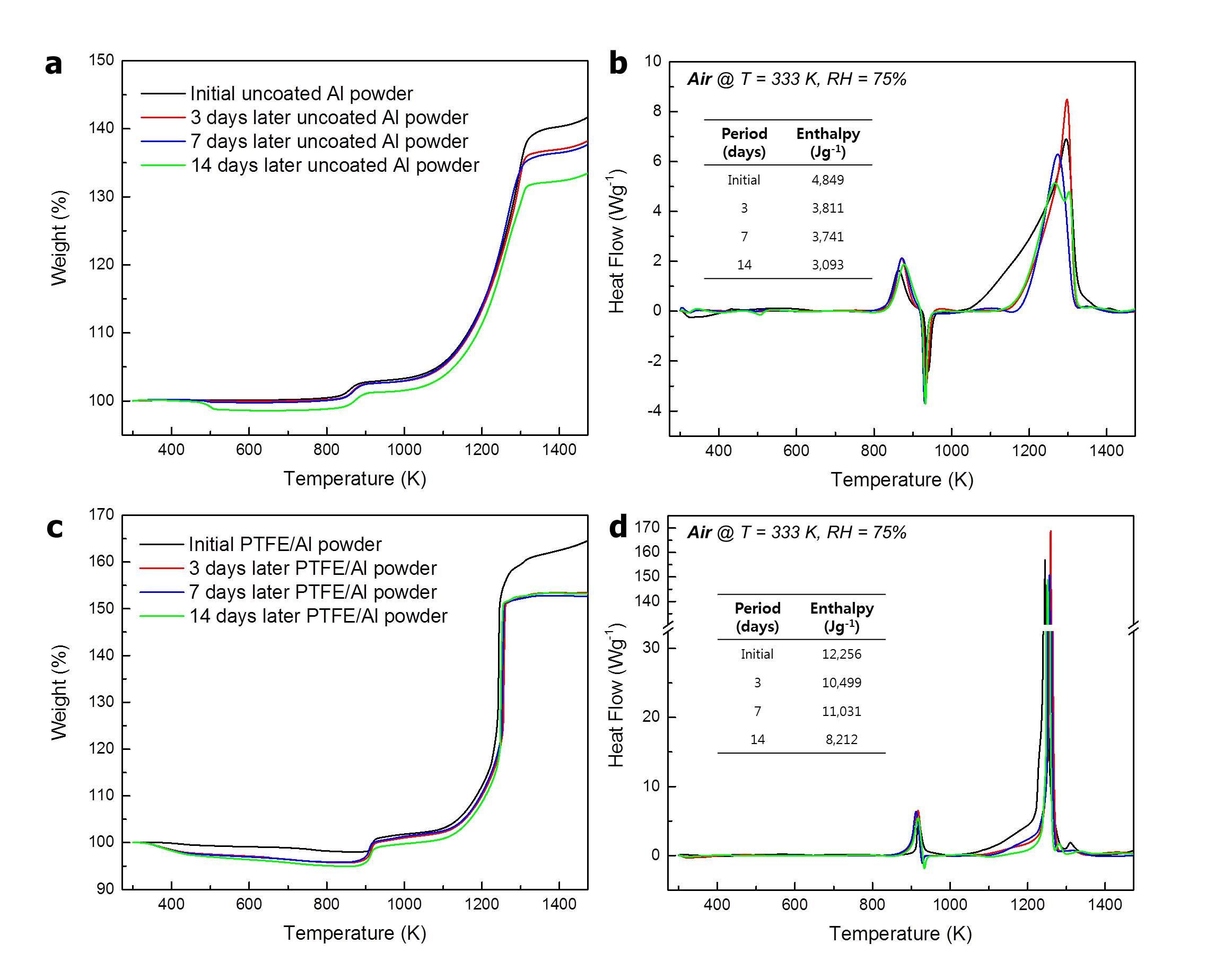


Figure S3. Comparison of the TGA/DSC curves of (a-b) uncoated Al and (c-d) PTFE/Al powders under air atmosphere over acceleration aging time.


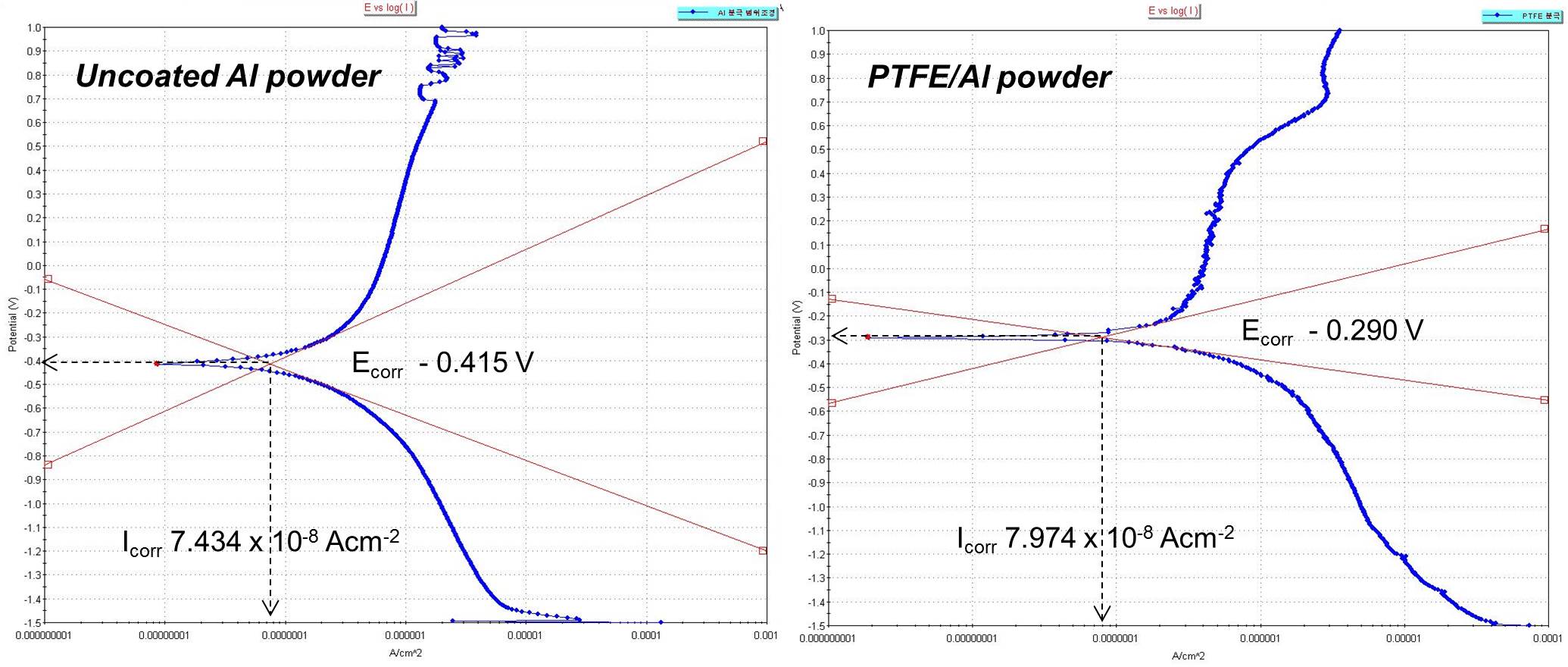


Figure S4. Potentiodynamic polarization curves with Tafel plot of uncoated Al and PTFE/Al powders.


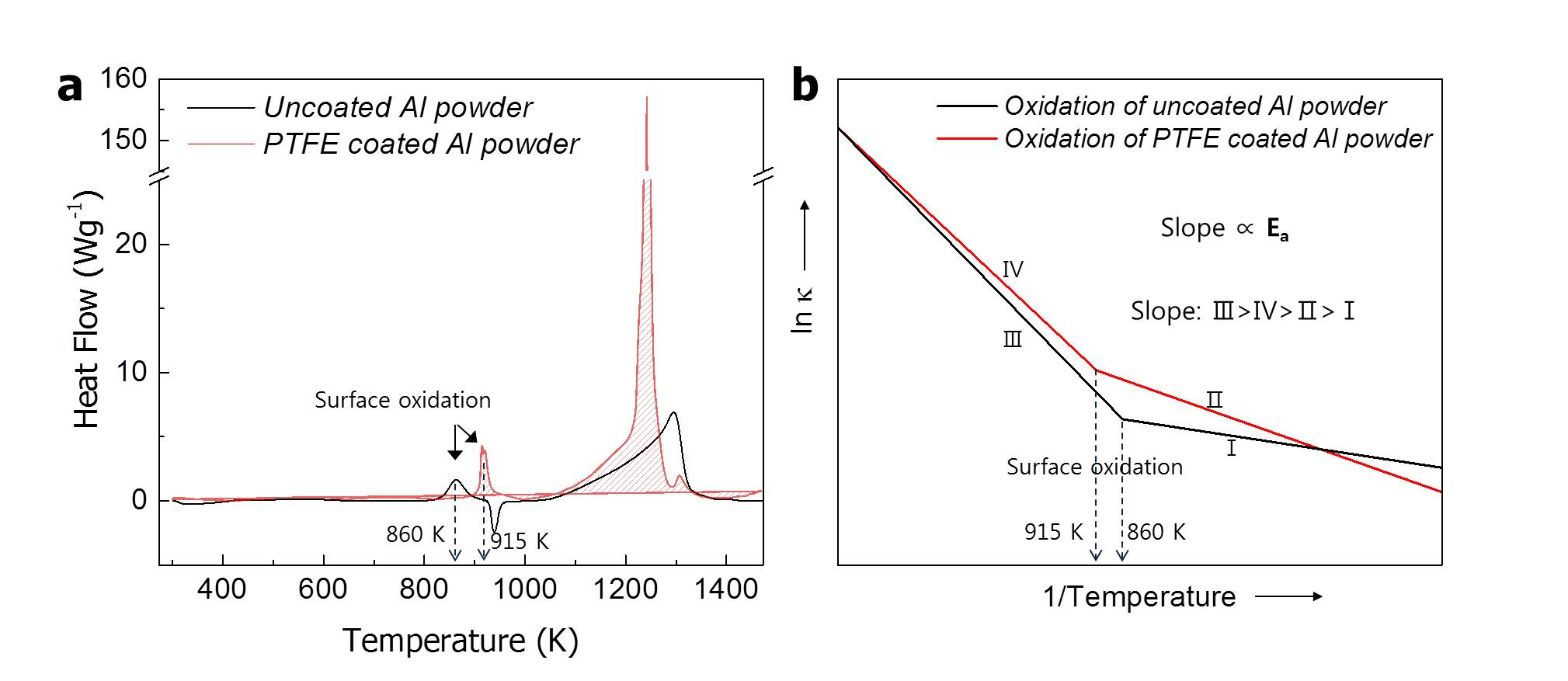


Figure S5. (a) DSC curves of uncoated Al and PTFE/Al powders and (b) Arrhenius plots of the both powders due to activation energy change during oxidation.

The Arrhenius equation can be given in the follow:

k = A exp (-E_a_/RT)

Taking the natural logarithm of Arrhenius equation yields:

ln k = -E_a_/R(1/T) + ln A

The slope of this linear equation is proportional to the activation energy. As shown in Figure S5a, since the PTFE/Al powder has higher activation energy than the uncoated Al powder, the surface oxidation starts later. In the case of main oxidation, it can be seen that the PTFE/Al powder starts a little faster. Therefore, the activation energy of the PTFE/Al powder postulated lower than uncoated Al powder after surface oxidation as shown in Figure S5b.


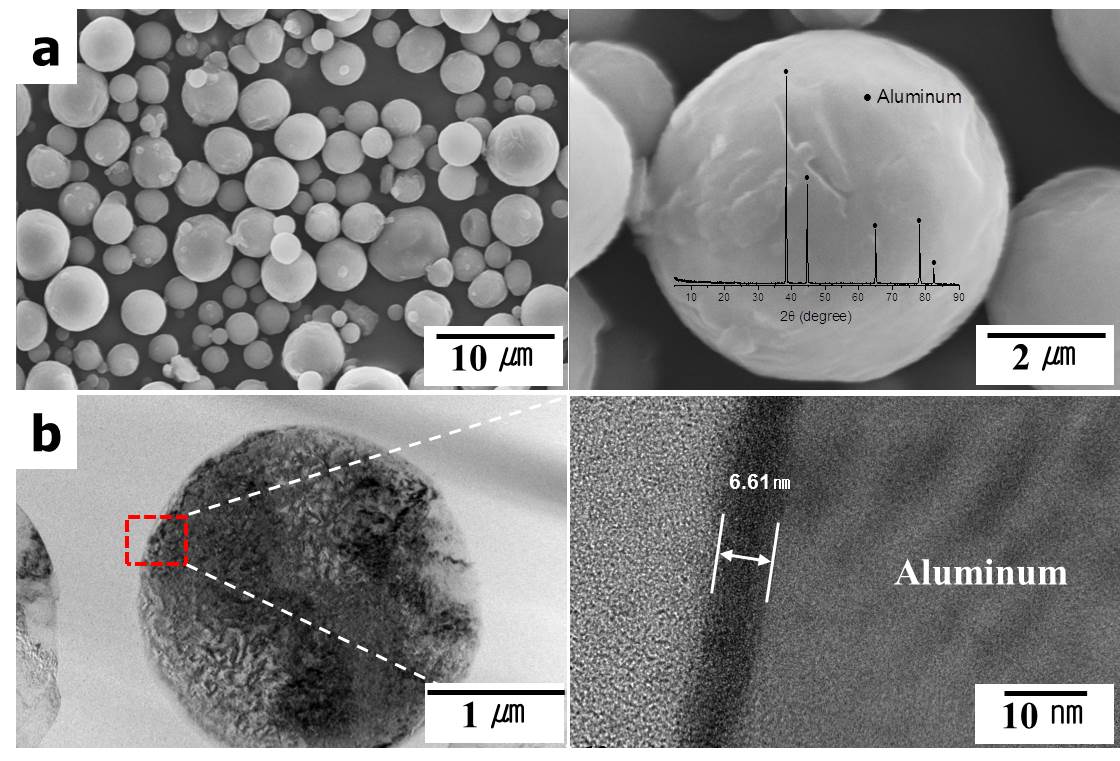


Figure S6. (a) SEM images and XRD pattern (right inset), (b) TEM images of uncoated Al particles used in this work.


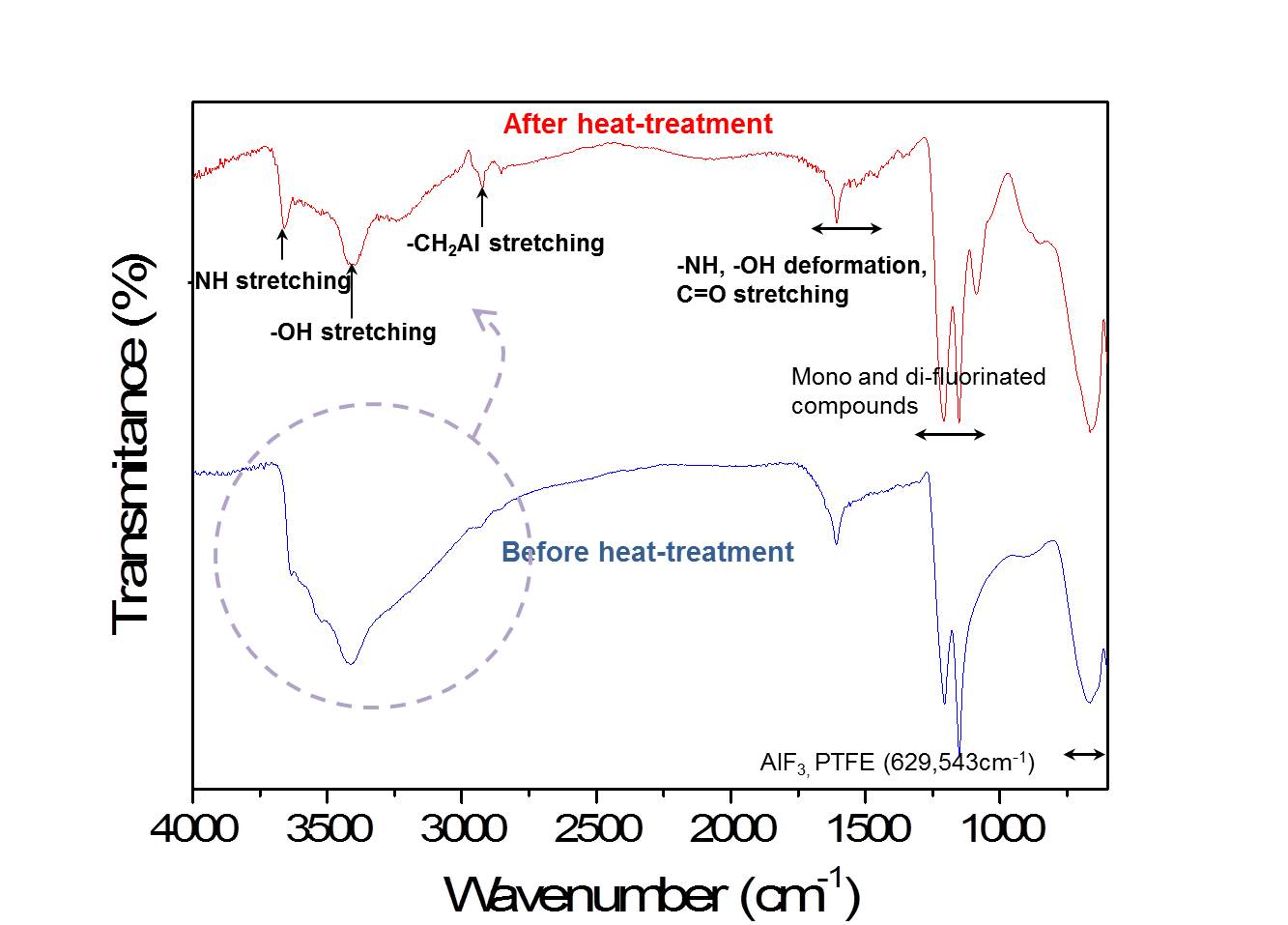


Figure S7. FT-IR spectra comparison before and after heat treatment of the prepared PTFE/Al powder.


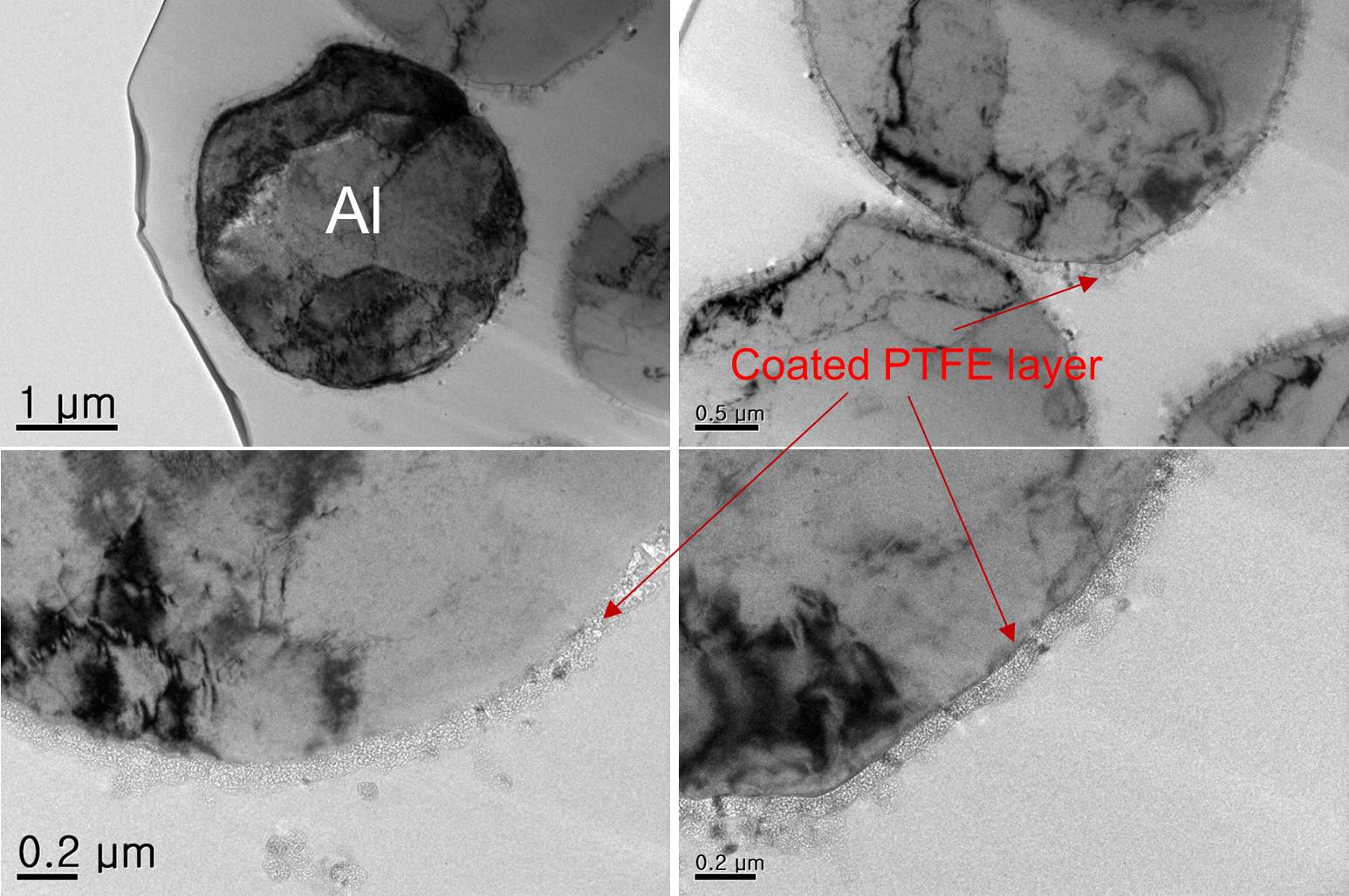


Figure S8. TEM image of a PTFE/Al particle specimen prepared using focused ion beam (FIB).

**Reference**

[1] M. Binnewies, E. Milke, *Thermochemical Data of Elements and Compounds,* Second, Revised and Extended Edition ed., Wiley-VCH, Weinheim, **2002**.
